# Double-Docking Technique, an Optimized Process for Intrathoracic Esophagogastrostomy in Robot-Assisted Ivor Lewis Esophagectomy

**DOI:** 10.3389/fsurg.2022.811835

**Published:** 2022-03-21

**Authors:** Fuqiang Wang, Hanlu Zhang, Guanghao Qiu, Zihao Wang, Zhiyang Li, Yun Wang

**Affiliations:** ^1^Department of Thoracic Surgery, West China Hospital, Sichuan University, Chengdu, China; ^2^West China School of Medicine, Sichuan University, Chengdu, China

**Keywords:** esophagus surgery, robotic surgery, anastomosis, esophageal carcinoma, Ivor Lewis esophagectomy

## Abstract

**Background:**

Though robotic Ivor Lewis esophagectomy has been increasingly applied, intrathoracic esophagogastrostomy is still a technical barrier. In this retrospective study, we introduced a double-docking technique for intrathoracic esophagogastrostomy to optimize surgical exposure and facilitate intrathoracic anastomosis. Moreover, we compared the clinical outcomes between the double-docking technique and anastomosis with a single-docking procedure in robotic Ivor Lewis esophagectomy.

**Methods:**

From March 2017 to September 2020, the clinical data of 68 patients who underwent robotic Ivor Lewis esophagectomy were reviewed, including 23 patients who underwent the double-docking technique (double-docking group) and 45 patients who underwent single-docking robotic esophagectomy (single-docking group). All patients were diagnosed with esophageal cancer or gastro-esophageal junction by biopsy before surgery. The technical details of the double-docking technique are described in this article.

**Results:**

There was no difference in the patient demographics data between the two groups. The median surgical time in the double-docking group was slightly shorter than in the classic group without statistical difference (380 vs. 395 min, *p* = 0.368). In the double-docking group, the median blood loss was 90 mL, the median number of lymph nodes harvested was 17, and the R0 resection rates were 100% (23/23). There were no differences in the surgical outcomes between the two groups.

**Conclusions:**

Based on our experience, the double-docking technique provides good surgical exposure when fashioning anastomosis, and such a technique does not increase the surgical time. Therefore, we believe that the double-docking technique is a safe and effective method for intrathoracic esophagogastrostomy while providing good exposure and ensuring the convenience and reliability of intrathoracic anastomosis.

## Introduction

Esophagectomy is one of the main methods to manage esophageal carcinoma (1). Ivor Lewis esophagectomy was firstly introduced in 1946 (2). In contrast to McKeown esophagectomy, the postoperative complications including anatomies leakage and recurrent laryngeal nerve paralyzes are remarkably decreased (3, 4). However, intrathoracic gastroesophagostomy is still the technical barrier of minimally invasive Ivor Lewis esophagectomy (5, 6). Recently, in favor of high-definition three-dimensional vision, tremor filtration, and an EndoWrist instrument, a surgical robot is thought to be perfectly suited to complex surgery in the narrow space (7–9). As a result, robotic Ivor Lewis esophagectomy (RILE) is increasingly applied to manage esophageal carcinoma (8).

Presently, the methods for robotic intrathoracic gastroesophagostomy are various, including circular stapling (10), linear stapling (5, 11), and hand-sewn applications (12). According to a recent review from Plat et al., circular stapling is considered the most appropriate method for robotic intrathoracic gastroesophagostomy (13). However, in most cases of robotic esophagectomy, the location of thoracic trocars does not benefit the insertion of a circular stapler and anastomosis. Firstly, sufficient space for creating a mini-thoracotomy in the chest wall is required to insert the circular stapler. In most cases of robotic esophagectomy, the thoracic trocars were located along the longitudinal axis of the patient (6, 10, 12, 14–19). Therefore, the wide surface area of the chest wall was covered by the trocars, which did not benefit the insertion of a circular stapler. Grimminger et al. suggested disconnecting the surgical robot to make space for insertion of a circular stapler when fashioning anastomosis (16). But this might be technically demanding. Secondly, the posterior wall of anastomosis cannot be checked under direct vision for the relative low position of the camera port in most cases of robotic esophagectomy. Egberts suggested repositioning the camera in the port closest to the back for superior overview in the da Vinci Xi platform (10). However, this change cannot be performed in all robotic platforms.

To solve the aforementioned problems, we optimize the anastomosis technique which needed two docking procedures in the thoracic phase of RILE. By using this double-docking technique, we can insert the stapler with a sufficient chest surface and check the posterior wall of anastomosis under direct vision when performing intrathoracic anastomosis. In this article, we describe the technical details of this double-docking technique and compare the clinical outcomes between this double-docking technique and esophagogastrostomy with the one-docking procedure we introduced previously in RILE (20, 21).

## Materials and Methods

### Patients

From March 2017 to September 2020, the clinical data of 68 patients who underwent RILE with intrathoracic end-to-side esophagogastrostomy were reviewed in this research. A total of 23 patients who underwent the double-docking technique (double-docking group) and 45 patients who underwent robotic esophagectomy with the single-docking procedure (single-docking group) in the thoracic phase of esophagectomy were included. Chest computed tomography (CT), abdominal CT, gastroscopic biopsy, and pulmonary function tests were routinely performed in all patients. Positron emission tomography–computed tomography (PET/CT) was also considered if necessary. All patients were diagnosed with lower esophageal carcinoma or esophagogastric junction carcinoma according to the results of preoperative gastroscopic biopsy. Patients who underwent robot-assisted McKeown esophagectomy or palliative esophagectomy were excluded. The demographic information and clinical data were collected from the hospital information system of West China Hospital, Sichuan University. The study protocol was approved by the Ethics Committee of West China Hospital of Sichuan University (2021-578). Written informed consent was obtained from all 68 patients.

### Operative Technique

#### Abdominal Phase

A four-arm da Vinci Si robotic platform (Intuitive Surgical, Inc.) was utilized. After general anesthesia, a double-lumen was intubated for two-lung ventilation in the abdominal phase. The patient was placed in the reverse Trendelenburg position and the patient cart was placed at the patient's head. Abdominal insufflation with CO_2_ was set at 12 mmHg. The abdominal port was placed in accordance with the method we introduced previously (22). A 12 mm camera port was firstly placed below the navel to guide the insertion of another four abdominal ports. The assistant port was placed at the right anterior axillary line below the costal arch and the port for robotic arm 3 was placed at the left anterior axillary line below the costal arch. The ports for robotic arms 1 and 2 were location at the midpoint of the line between the camera port and the port for robot arm 3 and between the camera port and assistant port, respectively. The left liver lobe was retracted with one 3/0 Prolene suture (Ethicon) as we previously introduced (23). Firstly, we ligated the left gastric artery and vein with Hem-o-lok clips and then transected the vessels with a harmonic scalpel. The lymph nodes around the common hepatic artery, celiac trunk, and origin of splenic artery were then removed. Thereafter, the short gastric vessels in the gastrosplenic ligament and gastrocolic ligament and the right gastric vessels were transected. Then, we made the gastric conduit intraperitoneally by using a linear stapler (ECHELON FLEX Powered Staplers 60 mm, Ethicon) introduced from the assistant port. Construction of the gastric conduit started from the pyloric antrum of the lesser curve. After firing approximately four to six reloads with the linear stapler along the greater curvature, a 4 cm-wide gastric conduit was eventually formed. Notably, the formation of the gastric conduit was not finished in the abdomen. The stapler line was reinforced with a continuous suture by using self-locking barbed sutures (3-0 Stratafix, Ethicon). Notably, the proximal stomach was still connected to the gastric conduit so than the gastric conduit could be pulled into the chest for the following procedure.

#### Thoracic Phase

**Figure 1:**
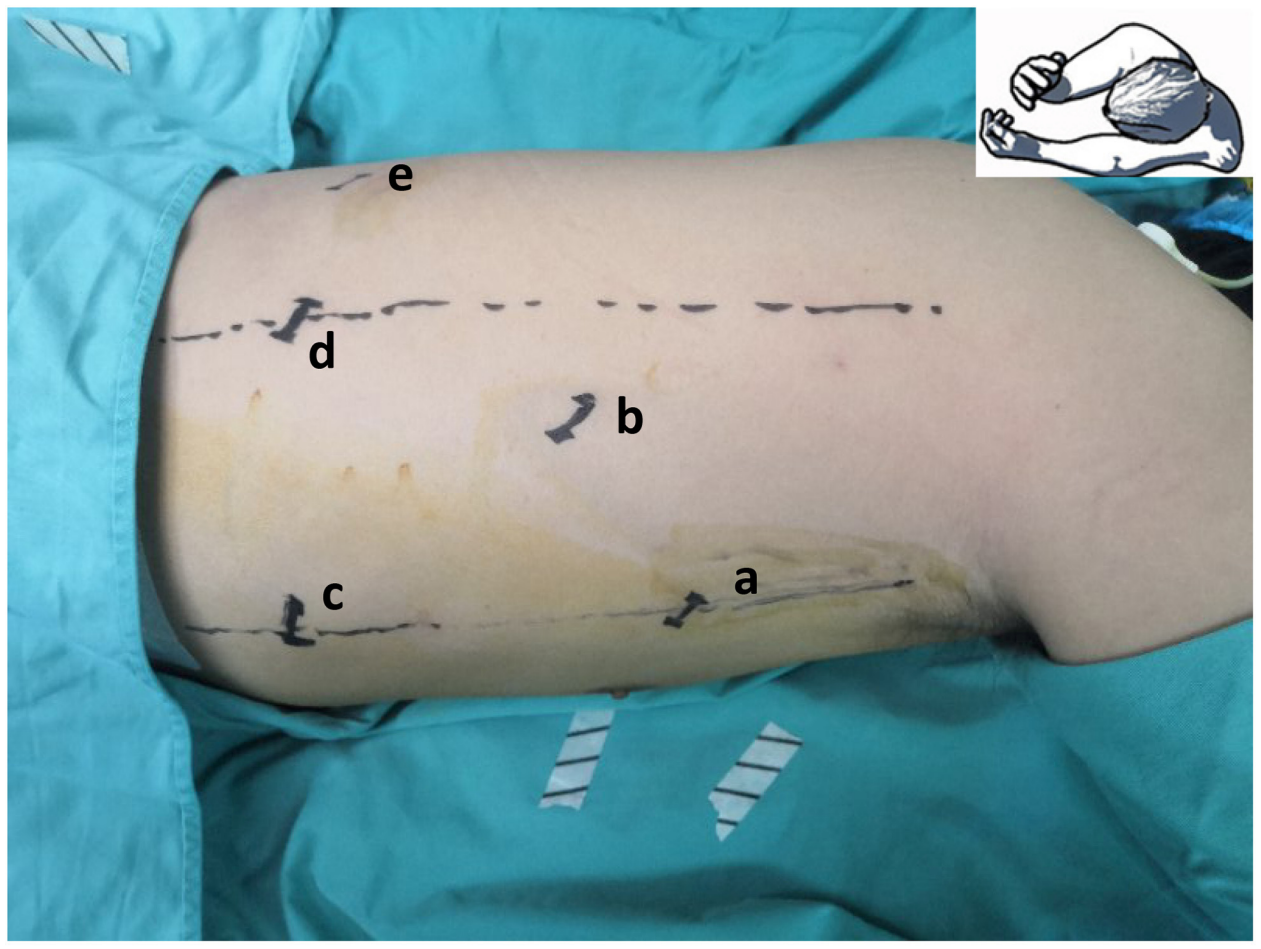
Location of thoracic port. “Port site a” located in the fifth intercostal space (ICS) at the midaxillary line, “port site b” located in the seventh ICS at the posterior axillary line, “port site c” located in the ninth ICS at the posterior axillary line, “port site d” located in the tenth ICS below the scapular tip, and “port site e” located 10 cm away from port site d in the tenth ICS.

Single-docking robotic esophagectomy was performed in accordance with our previous articles (20, 21). Here we present the technical details of the double-docking technique. The patient was repositioned in a left semi-prone position with single-lung ventilation. Thoracic insufflation with CO2 was set at 8 mmHg. As shown in Figure 1, “port site A” was located in the fifth intercostal space (ICS) at the midaxillary line, “port site B” was located in the seventh ICS at the posterior axillary line, “port site C” was located in the ninth ICS at the posterior axillary line, “port site D” was located in the tenth ICS below the scapular tip, and “port site e” was located 10 cm away from port D in the tenth ICS.

#### Esophageal Mobilization and Lymph Node Dissection (First Docking Stage)

**Figure 2:**
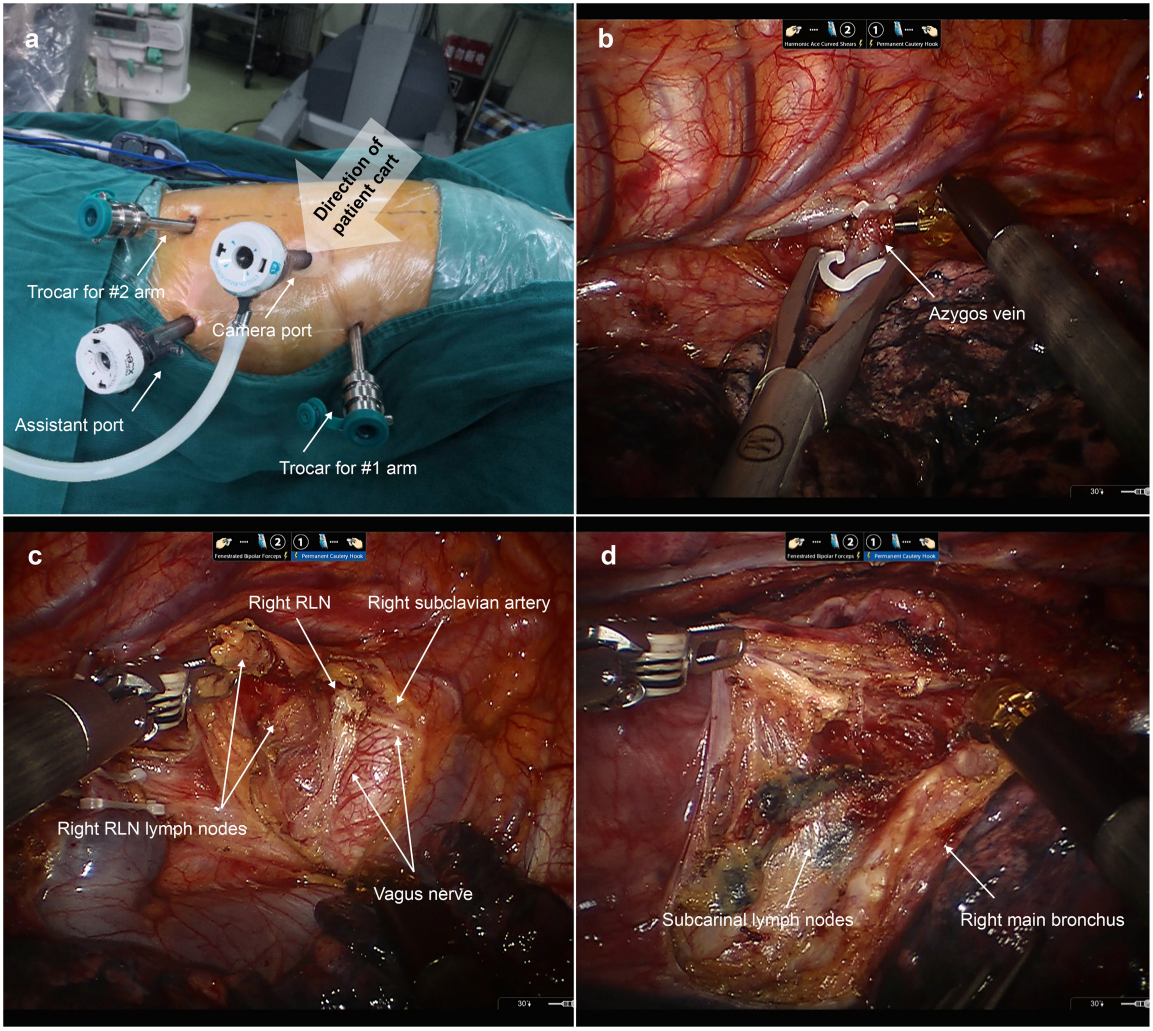
Esophageal mobilization and lymph node dissection during the first docking stage. **(a)** The location of thoracic ports applied in the first docking stage. A 12 mm trocar was placed at “port site B” as a camera port. Two 8 mm trocars were placed at “port site A” and “port site D” for robotic arms 1 and 2, respectively. Another 12 mm trocar was placed at “port site C” as an assistant port. **(b)** The azygos vein was ligated and then transected. **(c)** Dissecting the right recurrent laryngeal nerve (RLN) lymph nodes. **(d)** Dissecting the subcarinal lymph nodes.

As shown in Figure 2, in the first docking stage, a 12 mm trocar was placed at “port site B” as a camera port. Two 8 mm trocars were placed at “port site A” and “port site D” for robotic arms 1 and 2, respectively. Another 12 mm trocar was placed at “port site C” as an assistant port. The patient cart was positioned at the backside near the head of patient to ensure that the camera port, fourth thoracic vertebrae, and patient cart were located in the same line. We firstly ligated and then transected the azygos vein. Then, we started esophageal mobilization from the top of the thoracic esophagus to the esophageal hiatus with a harmonic scalpel. Dissection of thoracic lymph nodes was performed at the same time.

#### Esophagogastric Anastomosis (Second Docking Stage)

**Figure 3:**
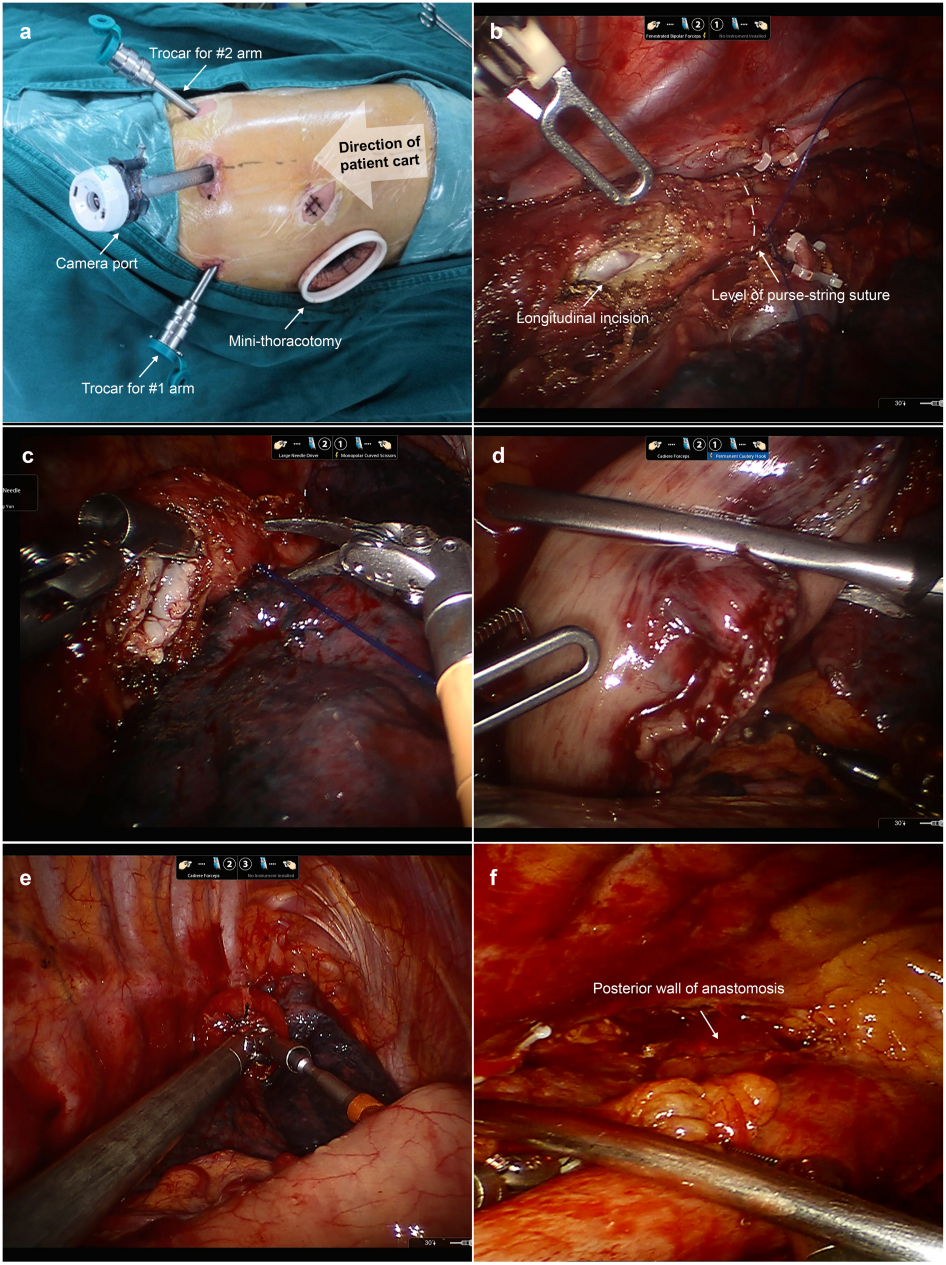
Esophagogastric anastomosis during the second docking stage. **(a)** The location of thoracic trocars and mini-thoracotomy applied in the second docking stage. The camera port was relocated in “port site D”. The trocars for robotic arms 1 and 2 were relocated at “port site C” and “port site E”, respectively. “Port site A” was extended to a 4 cm mini-thoracotomy in the fifth ICS. **(b)** A purse-string suture was placed to secure the esophagus around the anvil. A longitudinal incision in the esophagus was made 1 cm below the purse-string suture to insert the anvil. **(c)** We transected the esophagus with robotic scissors. **(d)** The gastric conduit and residual stomach were pulled up into the thoracic cavity. **(e)** The spike of the circular stapler was connected with the anvil with the help of Cadiere forceps. **(f)** The surgeon can check the posterior wall of anastomosis under direct vision.

After esophageal mobilization and lymph node dissection, we disconnected the da Vinci Si robotic system. As shown in Figure 3, the patient cart was repositioned at the patient's head. The camera port was relocated at “port site D”. The trocars for robotic arms 1 and 2 were relocated at “port site C” and “port site E”, respectively. The incision was extended to a 5 cm mini-thoracotomy in the fifth ICS and a disposable wound retractor was then inserted to protect the mini-thoracotomy. The anastomosis was performed upon the level of the azygos vein. First, a purse-string suture with a 3/0 Prolene suture was placed to secure the esophagus around the anvil. Afterwards, we cut open the esophagus 1 cm below the purse-string suture longitudinally with robotic scissors to insert the anvil of a 25 mm circular stapler (CDH stapler, Ethicon) in the lumen of the esophagus. Then, we transected the esophagus with robotic scissors at the level of the upper edge of the longitudinal incision. Notably, surgeons made sure that the whole layer of the esophagus was sutured and the mucosa was in the inner layer. Afterwards, the gastric conduit and residual stomach was pulled up carefully through the hiatus into the thoracic cavity. Then, the transected esophagus specimen, transected lesser curvature, and proximal gastric conduit was brought out of the chest through the mini-thoracotomy. After stapling with another two reloads of the linear stapler along the conduit stapler line, the specimen with lesser curvature was separated from the gastric conduit and the gastric conduit was completely created. Then, the circular stapler was inserted into the gastric conduit through an incision in the tip of the gastric conduit. The site near the greater curvature in the gastric conduit was chosen for anastomosis. The circular stapler in the lumen of the gastric conduit was then inserted in the thoracic cavity through the mini-thoracotomy. With the help of Cadiere forceps, the spike was connected to the anvil upon the level of the azygos vein. The gastric conduit was then stapled to the stump of the esophagus. So, the esophagogastrostomy was completed. The remnant gastric conduit was transected with the linear stapler and removed through the mini-thoracotomy. After the end-to-side anastomosis, we checked the anastomotic stoma under direct vision and reinforced the anastomotic stoma with barbed sutures. Lastly, a nasogastric tube was inserted under direct vision. The postoperative management was conducted in accordance with the method we previously introduced (5).

## Results

### Demographic Information

A total of 68 patients, including 23 patients who underwent the double-docking technique (double-docking group) and 45 patients who underwent robotic esophagectomy with the one-docking procedure (single-docking group) in the thoracic phase of esophagectomy. As shown in Table 1, there was no difference between groups in demographic information including age, gender, body mass index (BMI), American Society of Anesthesiologists (ASA) physical status classification, tumor location, TNM stage, histologic type, and comorbidity (*p* > 0.05).

**Table 1:** Demographic information of participants.

		**Single docking (*n* = 45)**	**Double docking (*n* = 23)**	***P*-value**
Age, y, median age (range)	63 (47–77)	59 (41–71)	0.17	
Gender				0.204
	Male	38 (84.4%)	22 (95.7%)	
	Female	7 (15.6%)	1 (4.3%)	
BMI, kg/m^2^,median BMI (range)		22.8 (16.3–30.0)	23.7 (16.5–29.9)	0.151
ASA				0.446
	Class 2	23 (51.1%)	14 (60.9%)	
	Class 3	22 (48.9%)	9 (39.1%)	
Tumor location				0.321
	Middle	2 (4.4%)	2 (8.7%)	
	Lower	36 (80.0%)	19 (82.6%)	
	GEJ	7 (15.6%)	2 (8.7%)	
pT stage				0.38
	Tis	1 (2.2%)	0	
	T1a	2 (4.4%)	0	
	T1b	1 (2.2%)	3 (13.0%)	
	T2	8 (17.8%)	6 (26.1%)	
	T3	32 (71.1%)	14 (60.9%)	
	T4a	1 (2.2%)	0	
pN stage				0.591
	N0	19 (42.2%)	10 (43.5%)	
	N1	16 (35.6%)	6 (26.1%)	
	N2	7 (15.6%)	4 (17.4%)	
	N3	3 (6.7%)	3 (13.0%)	
TNM stage				0.958
	0	1 (2.2%)	0	
	IB	3 (6.7%)	4 (17.4%)	
	II	1 (2.2%)	0	
	IIA	14 (31.1%)	6 (26.1%)	
	IIB	1 (2.2%)	0	
	IIIA	8 (17.8%)	3 (13.0%)	
	IIIB	13 (28.9%)	7 (30.4%)	
	IVA	4 (8.9%)	3 (13.0%)	
Pathology type				0.133
	SCC	36 (80.0%)	18 (78.3%)	
	AC	6 (13.3%)	1 (4.3%)	
	ASC	1 (2.2%)	0	
	NEC	0	1 (4.3%)	
	MANEC	1 (2.2%)	1 (4.3%)	
	Mixed NEC and SCC	0	2 (8.7%)	
	Mixed SCC and small cell carcinoma	1 (2.2%)	0	
Comorbidity				
	Diabetes mellitus	4 (8.9%)	2 (8.7%)	0.979
	Hypertension	9 (20.0%)	2 (8.7%)	0.244
	Coronary heart disease	1 (2.2%)	2 (8.7%)	0.253
	Cerebral infarction	1 (2.2%)	1 (4.3%)	0.63

*BMI, Body mass index; ASA, American Society of Anesthesiologists; GEJ, Gastro-esophageal junction; SCC, Squamous cell carcinoma; AC, Adenocarcinoma; ASC, Adenosquamous carcinoma; NEC, Neuroendocrine carcinoma; MANEC, Mixed adenoneuroendocrine carcinoma*.

### Perioperative Information

As shown in Table 2, the median surgical time was 395 min (range 290–480 min) for the single-docking group and 380 min (range 310–522 min) for the double-docking group. The median lymph nodes harvested was 19 for the single-docking group and 17 for the double-docking group (*p* = 0.944). There was no statistical difference between groups in surgical time, lymph node harvest, bleeding volume, drainage time, length of ICU stays, length of hospital stays, and postoperative complications. Postoperatively, anastomotic leakage happened in seven patients, including five patients (11.1%, 5/45) in the single-docking group and two patients (8.7%, 2/23) in the double-docking group. One patient in single-docking group died due to tracheoesophageal fistula 78 days after surgery. The anastomotic leakage of the other six patients was cured by drainage and/or irrigation without reoperation.

**Table 2:** Perioperative information of participants.

		**Single docking (*n* = 45)**	**Double docking (*n* = 23)**	***P*-value**
Surgical time, median min (range)		395 (290–480)	380 (310–522)	0.368
Bleeding volume, median ml (range)		100 (40–300)	90 (50–150)	0.183
Length of ICU stay, median day (range)		1 (0–23)	1 (0–3)	0.16
Length of hospital stay, median day (range)		12 (8–34)	11 (9–31)	0.25
LN harvest, median number (range)		19 (5–41)	17 (10–32)	0.944
Drainage time, median day (range)		8 (5–71)	9 (7–29)	0.414
Postoperative complications				
	Anastomotic leakage	5 (11.1%)	2 (8.7%)	0.757
	Pneumonia	5 (11.1%)	1 (4.3%)	0.369
	Wound infection	3 (6.7%)	0 (0%)	0.999
	TEF	1 (2.2%)	0 (0%)	1.000
R0 resection		44 (97.8%)	23 (100%)	1.000
In-hospital mortality		1 (2.2%)	0 (0%)	1.000

*TEF, Tracheoesophageal fistula*.

## Discussion

In this research, despite additional docking processes required in the double-docking method, we found the median surgical time for the double-docking group was even shorter than for the single-docking group (without statistical difference). Meanwhile, the rate of anastomotic leakage was also lower in the double-docking group (without statistical difference). Based on our experience on robotic esophagectomy, the double-docking technique offers the following advantages. First, in the second docking stage, the chest surface area is sufficient for inserting a circular stapler through the mini-thoracotomy. Second, the different locations of the camera port before and after the second docking ensured a favorable surgical exposure for esophageal mobilization, lymph node dissection, and anastomosis. Third, relocating the thoracic trocars for robotic arms avoided arm collisions when performing anastomosis. We assumed that it is the rational design of thoracic trocars and favorable surgical exposure that shorten the time for anastomosis and result in a lower median surgical time in the double-docking group.

First, the location of the mini-thoracotomy in the upper chest with a sufficient surface area benefits the insertion of a circular stapler in the double-docking technique. In most RILE, the camera port was designed to go in front of the scapula (10, 12, 18, 19). The camera port in front of the scapula might favor thoracic esophageal mobilization and lymph node dissection. However, based on our experience, the camera port located in front of the scapula was not conducive to the insertion of the stapler for limited chest surface when performing anastomosis. Inserting a circular stapler in such a narrow area may injure the gastric conduit and increase the risk of collision between robotic arms and the stapler. In our previously introduced single-docking technique for RILE, the mini-thoracotomy was located in the lower chest (at the seventh ICS in the posterior axillary line) due to limited surface in the upper chest (20, 21). However, the tip of the trocar for the camera might injure the gastric conduit when delivering the gastric conduit to the esophageal bed for anastomosis through the mini-thoracotomy in the lower chest. What is more, the camera needs to stay a long distance away from the anastomotic site to avoid injury to the gastric conduit by the tip of the trocar. Therefore, the surgical exposure was unfavorable when forming anastomosis in the single-docking technique. However, in the double-docking technique, the mini-thoracotomy was located in the upper chest and the camera was located in the lower chest. As a result, the gastric conduit can be delivered under direct vision so that the tip of the trocar for the camera may not injure the gastric conduit.

Moreover, relocation of the camera port in the second docking stage provided a superior overview and ensured a favorable surgical exposure when performing anastomosis. As we describe above, in RILE with the camera port located in front of the scapula, the surgical exposure was unfavorable for anastomosis due to the long distance between the camera and anastomotic site. What is more, the stapler may also obstruct vision to the anastomosis site when performing anastomosis, and the posterior wall of the anastomotic stoma cannot be checked under direct vision due to obstruction of the gastric conduit after anastomosis. In previous articles on RILE, some surgeons would check the anatomic leaks by utilizing blue dye (14) or instilled gas into the lumen of the reconstructed esophagus (17). But reinforcing the posterior walls of the anastomotic stoma was almost impossible due to the unfavorable exposure. In order to solve the vision problem, Grimminger et al. decided to disconnect the da Vinci robot system, and then the assistant would hold the camera to facilitate the anastomosis (16). But this change might be technically demanding. Egberts *et al*. suggested positioning the camera to the trocar closest to the back in da Vinci Xi platform to have a superior overview (10). The location of the camera port that Egberts applied was placed in accordance with the double-docking technique when performing anastomosis, which was located in the tenth ICS below the scapular tip. But in the da Vinci Si robotic system, the camera cannot be inserted through an 8 mm robotic trocar. Moreover, after positioning the camera to the trocar closest to the back, all the robotic arms were then located in the ventral side of the camera port, which might be difficult for the surgeon during anastomosis. Therefore, we decided to reposition the patient cart and applied “port site E” and “port site C” to the ventral and dorsal sides of the camera port to facilitate anastomosis, respectively.

Despite the requirement of two docking procedures, patient positioning did not need to change when performing anastomosis in RILE. Therefore, the time spent in the second docking process can be quite short for experienced assistants. In this research, the median surgical time in RILE with the double-docking process was 380 min, which is shorter than in RILE with a single-docking process. We determined that the favorable surgical exposure and rational design of the thoracic trocar shortened the time for anastomosis and thus shortened the surgical time.

Several limitations need to be noted regarding the present study. Firstly, we only compared the perioperative outcomes between groups lacking long-term follow-up information. Secondly, because this double-docking technique was newly adopted, the sample size of patients who underwent the double-docking technique was limited. Further study with prolonged follow-up time and larger sample size is required to further demonstrate the safety and feasibility of this double-docking technique. Notwithstanding these limitations, this preliminary study suggested that the double-docking technique was clinically safe and feasible to manage esophageal cancer for favorable exposure and improved convenience to perform anastomosis. Moreover, this double-docking technique can be performed in mainstream da Vinci robotic systems, such as S, Si, X, and Xi surgical systems. So, this technique has a high potential for popularization.

## Conclusion

In this study, we introduced a double-docking technique in RILE which can provide favorable exposure and improved convenience when performing thoracic esophageal mobilization, lymph node dissection, and anastomosis. Despite the fact that two docking processes were required, the median surgical time was not prolonged. Therefore, we believe that this double-docking technique is clinically safe and feasible to manage esophageal cancer for favorable exposure and improved convenience when performing anastomosis.

## Data Availability Statement

The raw data supporting the conclusions of this article will be made available by the authors, without undue reservation.

## Ethics Statement

The studies involving human participants were reviewed and approved by Ethics Committee of West China Hospital of Sichuan University. The patients/participants provided their written informed consent to participate in this study.

## Author Contributions

FW and HZ drafted the manuscript. YW, FW, and HZ designed the study, performed data interpretation, and participated in coordination. GQ, ZW, and ZL performed data collection and analysis. All authors read and approved the final manuscript.

## Funding

This study was supported by the 1·3·5 project for disciplines of excellence-Clinical Research Incubation Project, West China Hospital, Sichuan University (2021HXFH056), the grants from the Key Research Project of Sichuan Province (No. 2020YFS0249), and National Key Research Project of China (No. 2017YFC0113502).

## Conflict of Interest

The authors declare that the research was conducted in the absence of any commercial or financial relationships that could be construed as a potential conflict of interest.

## Publisher's Note

All claims expressed in this article are solely those of the authors and do not necessarily represent those of their affiliated organizations, or those of the publisher, the editors and the reviewers. Any product that may be evaluated in this article, or claim that may be made by its manufacturer, is not guaranteed or endorsed by the publisher.
